# Evolution of Electrochemical Cell Designs for In-Situ and Operando 3D Characterization

**DOI:** 10.3390/ma11112157

**Published:** 2018-11-01

**Authors:** Chun Tan, Sohrab R. Daemi, Oluwadamilola O. Taiwo, Thomas M. M. Heenan, Daniel J. L. Brett, Paul R. Shearing

**Affiliations:** 1 Electrochemical Innovation Lab, Department of Chemical Engineering, University College London, Torrington Place, London WC1E 7JE, UK; chun.tan.11@ucl.ac.uk (C.T.); sohrab.daemi.14@ucl.ac.uk (S.R.D.); o.taiwo@imperial.ac.uk (O.O.T.); t.heenan@ucl.ac.uk (T.M.M.H.); d.brett@ucl.ac.uk (D.J.L.B.); 2 Department of Earth Science & Engineering, Faculty of Engineering, Imperial College London, South Kensington Campus, London SW7 2AZ, UK

**Keywords:** X-ray tomography, electrochemical cell design, batteries

## Abstract

Lithium-based rechargeable batteries such as lithium-ion (Li-ion), lithium-sulfur (Li-S), and lithium-air (Li-air) cells typically consist of heterogenous porous electrodes. In recent years, there has been growing interest in the use of in-situ and operando micro-CT to capture their physical and chemical states in 3D. The development of in-situ electrochemical cells along with recent improvements in radiation sources have expanded the capabilities of micro-CT as a technique for longitudinal studies on operating mechanisms and degradation. In this paper, we present an overview of the capabilities of the current state of technology and demonstrate novel tomography cell designs we have developed to push the envelope of spatial and temporal resolution while maintaining good electrochemical performance. A bespoke PEEK in-situ cell was developed, which enabled imaging at a voxel resolution of ca. 230 nm and permitted the identification of sub-micron features within battery electrodes. To further improve the temporal resolution, future work will explore the use of iterative reconstruction algorithms, which require fewer angular projections for a comparable reconstruction.

## 1. Introduction

Energy storage devices have an increasingly significant role to play in all economic sectors with the de-carbonization of the global economy necessary to meet climate change goals. Rechargeable batteries are one of the key enabling technologies driving the shift to renewable energy with lithium-ion (Li-ion) battery technology becoming the mainstay of applications requiring high energy density such as portable electronics and electric vehicles (EVs). The Li-ion battery has been described as a rocking-chair battery where Li ions are intercalated into a transition metal oxide (TMO) or graphite host, respectively, during discharge and charge processes [1]. Beyond Li-ion technology, other lithium-based rechargeable batteries have been proposed including lithium sulfur (Li-S) and lithium air (Li-air) conversion-type chemistries. These offer theoretical capacities up to an order of magnitude higher and significantly higher energy densities compared to incumbent Li-ion cells [2]. However, their commercialization has been hindered by various design and engineering challenges imposed by mechanistic complexities.

While many competing cell chemistries—involving numerous redox pairs—exist in different stages of development, the basic architecture of the electrodes within most electrochemical devices involve some form of heterogeneous porous media on which electrochemical reactions occur. The microstructure of porous electrode materials often has a profound effect on the performance and lifetime of a cell [3]. Factors such as tortuosity and porosity of the electrodes influence the effective diffusivity of ions within the electrode and with the bulk of the electrolyte, contributing to cell impedance. The electrical conductivity of an electrode is also dependent on its microstructure in terms of the contact area between the different solid phases and is particularly important for cell chemistries involving electronically insulating active material. Heterogeneities within battery electrodes are known to contribute extensively to cell degradation since these induce local variations in current density and the state of charge.

Advances in synchrotron and laboratory-based radiation sources have led to X-ray based techniques such as X-ray absorption spectroscopy (XAS) [4], X-ray diffraction (XRD) [5], transmission X-ray microscopy (TXM) [6], and X-ray computed tomography (XCT) [7,8] becoming widely adopted to investigate the mechanisms behind the operation and degradation of electrochemical devices including lithium-based batteries. These complementary techniques span multiple time-scales and length-scales and provide information about the electronic states (XAS), crystalline states (XRD), and microstructures (TXM) of materials. Many beam and detector configurations exist, and this work will primarily be concerned with micro X-ray computed tomography (micro-CT) under full field illumination from an X-ray beam.

Due to the inherently heterogenous structures and processes occurring within batteries, there is a strong motivation to capture the physical and chemical states of their electrodes in 3D. Based on work on other porous media systems, ex-situ X-ray tomography has become widely adopted for the 3D characterization of battery electrodes to extract microstructural metrics indicative of battery performance including electrode tortuosity, porosity, pore, and particle size distributions [3,9,10,11].

In addition to these metrics, various authors conducted post mortem micro-CT and nano-CT studies to understand the factors contributing to electrode degradation. Furthermore, post Li-ion technologies typically involve conversion-type chemistries with liquid state or alloying reactions where significant changes typically occur (e.g., large volume change during lithiation of Si anodes) [12]. In these systems, tomographic measurements are of even greater interest in improving the mechanistic understanding of these inherently three-dimensional processes.

The development of in-situ electrochemical cells along with recent improvements in radiation sources have expanded the capabilities of micro-CT as a technique for longitudinal studies: the ability to track the same spatial volume of electrode as a function of some variable effectively eliminates variability inherent in ex-situ studies [13,14,15]. However, considerable challenges exist in the optimization of in-situ cell designs to achieve spatial and temporal resolutions compatible with the phenomena of interest. In this case, we aim to present an overview of the capabilities of the current state of technology and demonstrate novel tomography cell designs we have developed to push the envelope of spatial and temporal resolution whilst maintaining fidelity to the electrochemical performance of larger format cells.

## 2. Results and Discussion

The suitability of an in-situ electrochemical cell design for X-ray characterization is largely dependent on size, geometry, and materials used in its construction. Within the scope of lab-based research environments, cells used for pure electrochemical characterization of battery materials take the form of coin, pouch, and cylindrical geometries. X-ray transparent materials (i.e., polyimide) have been introduced to coin and pouch cells by numerous authors for in-situ and operando spectroscopic or imaging applications [16,17,18] in attempts to reduce the interference of ancillary components in the X-ray beam path. However, in the quest for a finer resolution, highly specialized cell designs are essential to achieve an acceptable signal-to-noise ratio within reasonable acquisition times because of limitations such as flux and detector sensitivity in imaging systems. While operando cell designs often come at the expense of electrochemical performance, the wealth of information obtained from these advanced characterization techniques vastly outweighs the higher cell impedances and lower achievable capacities. Acquisition times are even more important for tomography, which requires the collection of adequate projections at sufficiently fine angular increments to reconstruct. Some parameters that need to be optimized will be discussed in a later section.

The ideal sample for tomographic reconstruction is one that fits fully within the detector field of view (FOV) through all radiographic projections: samples larger than the FOV such as coin and pouch cells will result in the truncation of sinogram data. The horizontal resolution of charged-coupled device (CCD) or flat panel detectors do not typically extend beyond 2048 pixels (equivalent to a FOV of ca. 2 mm at 1 µm pixel size) and it is desirable to fit as much of the cell within the FOV to mitigate out of field artefacts present in interior tomographies. Cylindrical Swagelok-type cell designs [14] ensure attenuation lengths that are uniform on average for all projections as well as a weakly attenuating cell body and are highly desirable for in-situ characterization. A comparison of the properties of common materials used in the construction of Swagelok-type cells is presented in Table 1.

### 2.1. Optimization of Tomography Parameters

Imaging parameters for in-situ tomography acquisition are highly dependent on both the sample and instrument configuration and have to be optimized to produce the best possible image and resolution within a reasonable acquisition time. For lab-based instruments, a critical constraint imposed on the design of in-situ cells is sample size since this dictates the exposure time. For high-resolution micro-studies, this is typically approximately ca. 1 min per radiographic projection because of the limited brilliance of lab-based X-ray sources. The variables that have to be considered are discussed below.

### 2.2. X-ray Beam Energy and Its Effect on Intensity and Transmission

When X-rays interact with a sample, the intensity of the incident beam is attenuated by the sample and the ratio of the transmitted, *I*, to the incident beam intensities, *I*_0_, is known as transmission, *T*, and is defined by the equation below.

(1) $$T=\;\frac{I}{I_{0}}$$

For a monochromatic beam of known energy, transmission is related to the linear attenuation coefficient, *μ*, and thickness of a material, *t*, by the exponential relation known as the Beer-Lambert law.

(2) T=exp(−µt)

The linear attenuation coefficient, *μ*, is a function of the density of the material *ρ*, total cross-section per atom, *σ_tot_*, and the atomic mass of the element of interest, *A_r_*, displayed in Equation (3), which is defined by Hubbell [19].

(3) $$\frac{\mu }{\rho }=\sigma_{tot}\frac{N_{A}}{A_{r}}$$

where the total cross-section per atom, *σ_tot_*, accounts for each contribution from the principal photon interactions with the material [20] and *N_A_* is the Avogadro constant. Thus, the linear attenuation coefficient, corresponding to material composition and density, can be reconstructed in three-dimensional space with the appropriate inversion algorithm such as the inverse Radon transform. In practice, this is complicated by the use of polychromatic radiation produced by most lab X-ray sources and, in the absence of calibration with a phantom of known composition, micro-CT is most often used to inspect the microstructure within a material with some a priori knowledge of the composition of the sample.

In lab X-ray sources, the intensity and spectrum of the X-ray beam can be controlled by adjusting the X-ray tube voltage and current (i.e., source energy) and by changing the target material. Elements such as Cr, Co, Cu, Mo, Ag, and W are commonly used X-ray targets which each have their own characteristic spectra. In addition to the characteristic emissions of the X-ray target, a broad spectrum of X-rays is emitted via Bremsstrahlung radiation, up to a peak photon energy equivalent to the X-ray tube voltage and a polychromatic beam is produced. While an increase in the X-ray tube voltage and/or current will result in an increase in both incident and transmitted intensities, transmission (i.e., the ratio between incident and transmitted intensities) is not a function of tube current and increases only with tube voltage.

Barring discontinuities in the attenuation coefficients of elements at photon energies close to their specific absorption edges, an increase in mean photon energy will result in an increase in transmission as more photons reach the detector without interacting with the sample. Transmission is a critical variable that influences image quality and Reiter et al. have found that, for an ideal detector, ca. 14% transmission results in the most optimal signal-to-noise ratio [21]. In a multi-component system containing phases of very different attenuation coefficients, a compromise has to be made when selecting the beam energy. This is particularly acute when imaging battery electrodes since transmission varies greatly between the highly attenuating active material particles (consisting of transition metal oxides for Li-ion positive electrodes) and the weakly attenuating carbon and binder phase, which was discussed previously [22].

Another important acquisition parameter is the exposure time per projection since sufficient detector counts are necessary to form a low noise image depending on the dynamic range of the detector. Detector counts are proportional to the transmitted intensity integrated over the exposure time through the image formed on the scintillator. Exposure time is largely independent from transmission and has to be optimized by taking into account two opposing variables: adequate signal-to-noise ratios (long exposure) and minimized blurring induced by sample motion and thermal drift (short exposure).

**Figure 1:**
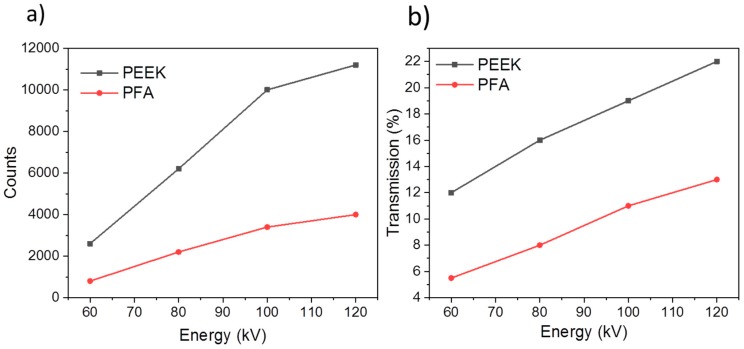
(**a**) Detector counts and (**b**) transmission as a function of source voltage for a 60 s exposure time through an NMC111 electrode in the PFA and PEEK cell.

To determine the optimal acquisition parameters for the PFA and PEEK cells, radiographs were acquired from both cells containing NMC111 electrodes in a half-cell arrangement over a range of X-ray source voltages. Line profiles were drawn across the electrode layer in the radiographs to obtain the graphs presented in Figure 1. Manufacturer’s specifications for the ZEISS Xradia Versa 520 laboratory micro-CT instrument used suggested at least 5000 counts and, as seen in Figure 1, this is unachievable even at 120 kV. On the other hand, a factor of ca. 3 improvement in counts is observed with the PEEK cell. Furthermore, transmission across the PEEK electrode at ca. 70 to 80 kV is optimal at around 14%, which indicates that the resulting reconstruction will likely have a good signal-to-noise ratio.

### 2.3. Number of Projections

The Nyquist-Shannon theorem can be applied to determine the angular resolution or an equivalent number of projections, which is theoretically required for reconstruction. The theorem states that an object has to be sampled with a frequency greater than twice the highest frequency of the features within the object. For a comprehensive mathematical treatment of sampling conditions for various beam geometries, the reader is directed to texts such as those by Natterer [23], Epstein [24], or Herman [25]. Zhao et al. suggested a general rule of thumb for cone beam CT where the number of projections, *N_proj_*, should be spaced to ensure the angular separation between each projection at the edge of the field of view (*FOV*) is equivalent to the voxel size, *b_vox_*.

(4) $$N_{proj}\geq \frac{2\pi }{\arctan\left(2b_{vox}/FOV\right)}$$

As described earlier, the sample diameter is likely to exceed the detector size for in-situ cells and the theoretical number of projections becomes a function of the detector size: a 2K detector with 2048 pixels will require the acquisition of ca. 6400 projections. In reality, it is unlikely for the spatial frequency of features within the sample to exceed the sampling frequency and fewer projections may be acquired without compromising image quality [26].

### 2.4. Magnification and Resolution

There are two sources of magnification in a conventional laboratory micro-CT without X-ray optics: geometric and optical magnification. Micro-focus sources with a small spot size produce a divergent cone beam that provides geometric magnification. The image formed on the scintillator can also be magnified through the use of objective lenses. On the detector, counts can be improved at the expense of resolution by combining neighboring pixels in a process called binning.

The pixel size achieved after pixel binning and magnification must be capable of capturing the phenomena of interest at a representative spatial resolution. For example, if the cell geometry and the alignment of its components are to be observed, a lower magnification and a higher binning may be appropriate while, for electrode-level phenomena and detailed microstructure, a higher magnification and the lowest possible binning should be used. 

### 2.5. Optimization of Battery Electrodes Used for In-Situ Cells

Most commercial Li-ion batteries utilize a ‘full-cell’ configuration consisting of a transition metal oxide positive electrode paired with a graphite negative electrode [27] and containing porous networks where Li-ions can reversibly intercalate during charge and discharge processes. Commercially relevant positive electrode materials include Lithium Cobalt Oxide (LiCoO_2_–LCO), Lithium Manganese Oxide (LiMnO_4_–LMO), and Lithium Nickel Manganese Cobalt Oxide (LiNi_0.33_Mn_0.33_Co_0.33_O_2_, NMC111), which is a potential complicating factor for in-situ cells due to the relatively dense and attenuating nature of these materials.

The in-situ cells presented in this paper were mainly prepared in a ‘half-cell’ arrangement where the electrode of interest is paired with a lithium metal negative electrode instead. This arrangement is commonly used in materials research since the specific electrochemical performance and practical voltage range of the electrodes of interest can be decoupled. Electrochemical parameters include the voltage range and C-rate that a battery is subject to and are important factors that influence the rate of degradation. A C-rate of 1C is equivalent to the current that will charge or discharge the entire capacity of a battery in an hour and can be calculated from the specific capacity, active material loading, and the mass or diameter of an electrode.

The electrodes, electrode arrangement, and cell environment have to be carefully optimized to ensure performance comparable to larger format cells, which is a non-trivial task given the conflicting need to minimize cell dimensions for micro-CT. Some factors that have to be considered include: electrode alignment and distance between electrodes that determines the ionic resistance across the electrolyte, compressive forces within the cell that controls the contact resistance between the electrode current collectors and plungers, and dead volume within the cell that influences electrode wetting due to gas evolution. Thus, the iterative approach, as presented in this work, was necessary to optimize electrochemical performance.

### 2.6. Evolution of Cell Designs

In most cases, region of interest (ROI) tomographies are carried out since the sample size is much larger than the detector size. Thus, grayscale data obtained in the reconstruction process does not correspond directly with linear attenuation coefficients even though discontinuities are captured. In this section, we explore the evolution of in-situ cell designs for electrochemical control.

#### 2.6.1. In-Situ Coin Cell

**Figure 2:**
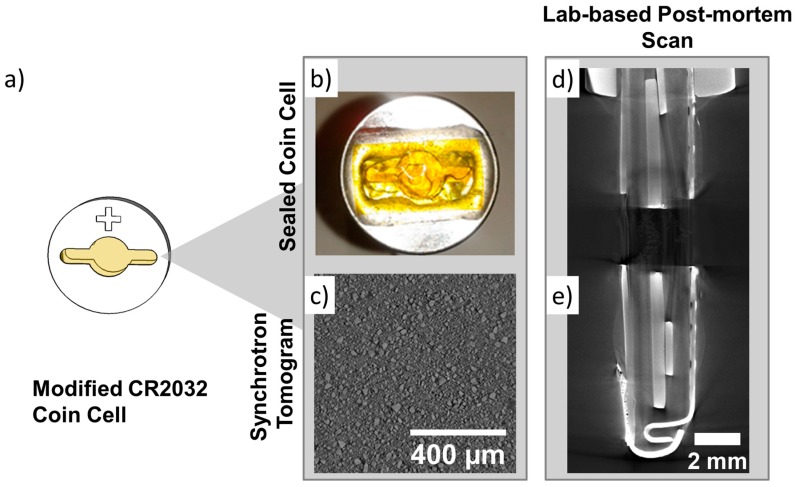
(**a**) Modified CR2032 coin cell rendering and (**b**) image of the Kapton window attached to the cell. (**c**) Reconstructed slice of a LiMnO_2_ electrode acquired at a synchrotron facility and (**d**,**e**) post-mortem CT scan of the entire cell showing its components.

The initial iteration of in-situ tomography cells we developed consisted of a modified coin cell with a Kapton window, which is illustrated in Figure 2. In this geometry, angular projections were acquired with a planar scan trajectory (i.e., with centre of rotation parallel to the current collector of the electrode). The Kapton window provided an angular range of 147° through which the X-ray beam could pass through unobstructed by the coin cell casing.

The X-ray transparent window significantly minimizes attenuation of the X-ray beam by the stainless steel casing, which reduces imaging artefacts such as beam hardening observed in Figure 2d. In-situ cells in this geometry were tested with both laboratory and synchrotron micro-CT instruments and appear to yield better imaging results with the latter. With synchrotron micro-CT, local tomograms with a voxel size of ca. 0.365 µm were obtained as opposed to larger voxel sizes (up to 2 µm) with laboratory micro-CT. This is due to limitations in achievable geometric magnification caused by the size of the in-situ cell. Whilst some reconstruction artefacts are to be expected due to the truncated angular range of the projections, which is shown in Figure 2c, image quality obtained with synchrotron micro-CT is remarkably comparable to ex-situ micro-CT scans of the same electrode material where the full angular range was captured.

**Figure 3:**
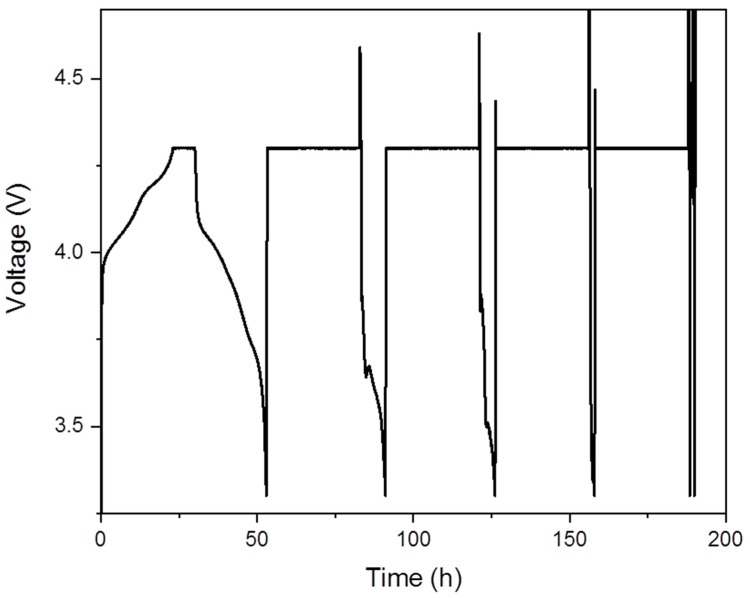
Voltage profile of in-situ windowed coin cell cycled at C/20 in constant current-constant voltage mode.

The main drawback experienced with the in-situ coin cell design was regarding the electrochemical performance and stability shown in Figure 3. The cell was cycled in constant current-constant voltage mode at a rate of C/20 and achievable capacity is comparable to standard coin cells for the first ca. 50 h beyond which cell capacity rapidly deteriorates. As outlined in Table 1, moisture impermeability is crucial for electrochemical performance and stability because Li-ion electrolytes are highly sensitive to moisture. Although materials such as Kapton and Mylar have been used extensively as X-ray transparent materials, we hypothesize that the poor stability of the cell was due to moisture absorption through the Kapton window. This was evident in white deposits that formed in the cell over time after exposure to ambient conditions.

#### 2.6.2. In-Situ Swagelok-Type PFA Cell

**Figure 4:**
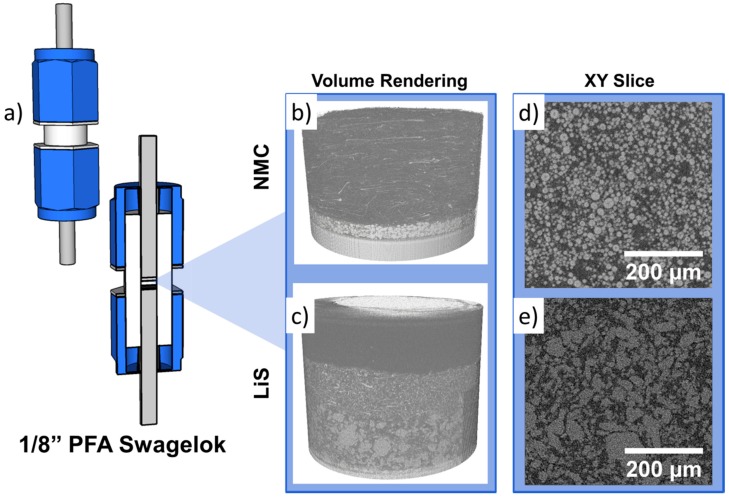
(**a**) Rendering of the 1/8” PFA Swagelok in-situ cell. (**b**) Volume rendering of NMC and (**c**) Li-S electrodes and the (**d**,**e**) their respective virtual slices.

The electrochemical stability issues faced in the in-situ coin cell design resulted in the need to develop a cell capable of extended cycling and yet remaining easy to assemble and suitable for X-ray imaging. Inspired by larger Swagelok-type cells described by previous authors [28], we have modified 1/8” PFA Swagelok straight unions to be used for in-situ X-ray characterization, which is illustrated in Figure 4a. Consisting of stainless steel plungers on both sides in contact with the positive and negative electrodes, the cell is mounted upright and imaged with a cylindrical scan trajectory. By virtue of this design, the cell body in the beam path is thinned down and no highly attenuating phases (such as stainless steel) enter the field of view of the entire cell stack during tomography, which reduces undesirable artefacts significantly. Furthermore, the rotational symmetry of the cell ensures compatibility with laboratory micro-CT where projections have to be acquired for the full 360° due to the cone beam nature of the X-ray source [29].

**Figure 5:**
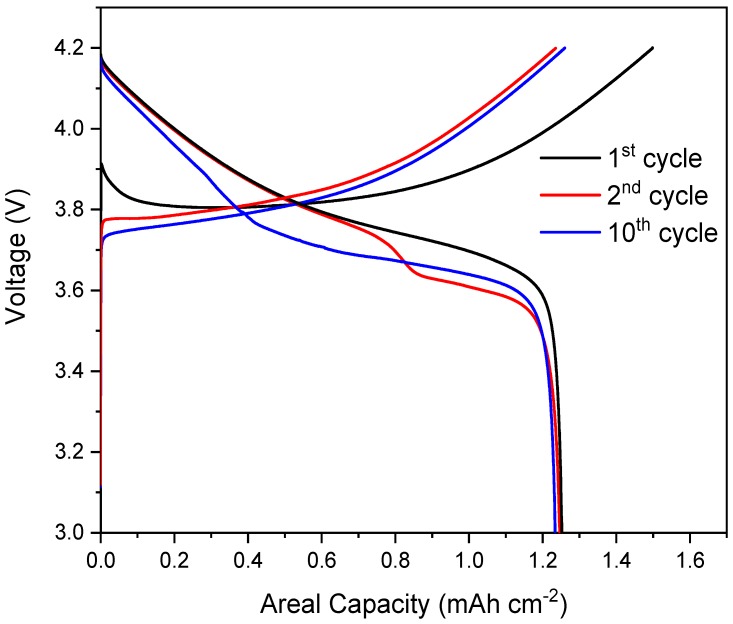
Charge and discharge curves for the 1st, 2nd, and 10th cycles for the NMC cell.

Li-ion half-cells with NMC as a positive electrode were constructed in the PFA in-situ cell and exhibited excellent electrochemical stability over numerous cycles, as presented in Figure 5, with virtually no capacity degradation across 10 cycles and achieved an areal capacity of ca. 1.25 mAh cm^−2^.

While the PFA cell design exhibits excellent electrochemical stability, the diameter of the resulting cell (ca. 10 mm) is relatively large and, therefore, more suited to synchrotron micro-CT where beam brilliance is not a limiting factor. As shown in Figure 4e, where we have previously reported an in-situ study of Li-S cells [13] with laboratory micro-CT, tomograms are relatively noisy. Acquisition times in laboratory micro-CT may be about ca. 48 h at high magnifications since long exposure times are required to achieve an adequate signal-to-noise ratio. Furthermore, polymers such as PFA and PTFE are also known to degrade from exposure to radiation, which turns brittle as a function of the total X-ray dose.

#### 2.6.3. In-Situ PEEK Cell

**Figure 6:**
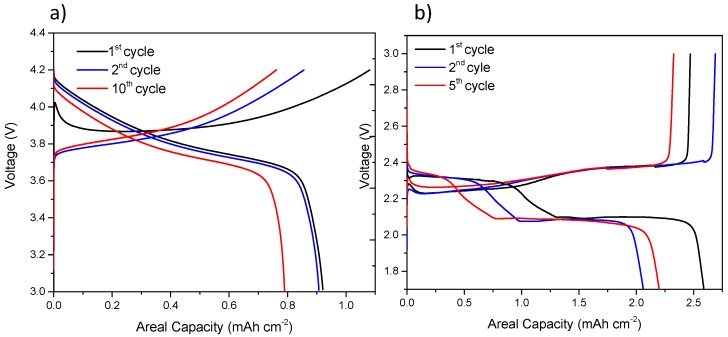
Electrochemical cycling data of (**a**) Li-ion half-cell with NMC positive electrode and (**b**) Li-S cell with elemental sulfur electrode.

To improve radiation resistance and further reduce sample size to improve image quality and acquisition times in laboratory micro-CT, a bespoke PEEK cell was developed. PEEK was selected since it is compatible with common Li-ion electrolytes and is non-reactive with metallic lithium. The PEEK cell has a comparable geometry to the PFA cell with a factor of 4 reduction in electrode diameter from ca. 3.2 mm to ca. 0.8 mm. For comparison, the same NMC material was cycled in the PEEK cell and the electrochemical performance, shown in Figure 6a, is similar to the PFA cell even though some capacity loss occurs after 10 cycles. A Li-S cell was also constructed with the same PEEK cell with electrochemical data presented in Figure 6a. Despite the marginally poorer electrochemical performance and stability of the PEEK cell compared to the PFA cell due to the smaller electrode diameter, this is a reasonable compromise considering the improvements in spatial and temporal resolution achievable with this cell design.

**Figure 7:**
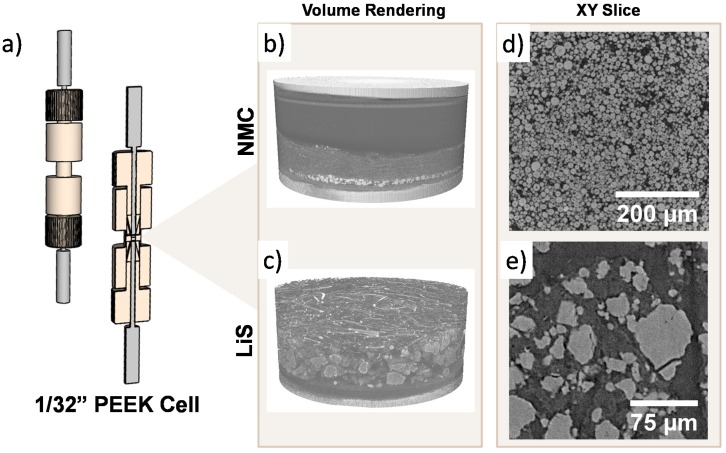
(**a**) Rendering of the 1/32” PEEK cell. (**b**) Volume rendering of NMC acquired at 20× magnification and (**c**) Li-S electrodes acquired at 40× magnification and the (**d**,**e**) their respective virtual slices.

The total attenuation due to the cell body and electrode is decreased with a reduction in the diameter of the in-situ cell, which leads to shorter acquisition times in laboratory micro-CT. Furthermore, it is desirable to minimize the source-sample and sample-detector distances in laboratory micro-CT to reduce attenuation by air and the profile of the PEEK cell is highly suited to this. Relevant information is shown in Figure 7a.

A tomogram of the Li-S PEEK cell was acquired at 40× magnification with a laboratory micro-CT instrument and a virtual slice of the sulfur electrode is shown in Figure 7e along with the associated volume rendering in Figure 7c. The volume rendering in Figure 7b shows the different layers present within the cell with lithium metal on top, glass fibre separator in the middle and NMC electrode below, which is sandwiched between two current collector plungers. In Figure 7c, the higher magnification enables the electrode to be visualized in greater detail at the expense of FOV. Most significantly, a voxel size of ca. 230 nm was achieved, which permits the identification of sub-micron features within the electrode, as shown in Figure 7e, with an adequate signal-to-noise ratio and a relatively fast acquisition time due mainly to the diminutive electrode size of the in-situ PEEK cell. In addition to the relatively featureless and X-ray transparent PEEK cell body, the smaller electrode diameter leads to a reduction of complex geometries external to the field of view, and, in turn, fewer artefacts after reconstruction. Furthermore, the radiation resistance of PEEK also expands the possibilities for long-term micro-CT studies on battery degradation to be carried out in-situ. For instance, this includes the investigation of particle cracking within Li-ion positive electrode materials after a large number of cycles.

### 2.7. Improvement in Image Quality and Electrochemical Performance through In-Situ Cell Optimization

**Figure 8:**
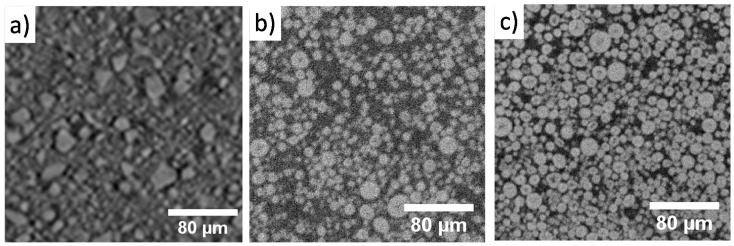
Improvement in image quality between (**a**) coin cell scan performed at a synchrotron facility for an LMO electrode, (**b**) Swagelok-type PFA cell, and (**c**) PEEK cell scans performed with a laboratory micro-CT instrument, Zeiss Xradia Versa 520 for an NMC electrode.

Improvements in image quality over the iterations of in-situ cell design are demonstrated in Figure 8 and, while these improvements cannot be quantified due to the combination of synchrotron and laboratory CT used, in-situ studies that were once exclusive to synchrotron sources (Figure 8a) are now within the realm of capability of laboratory micro-CT instruments. It is expected that the marked improvement in image quality between the PFA cell shown in Figure 8b and PEEK cell in Figure 8c will also translate to synchrotron studies due to the reduction of material external to the FOV. This is advantageous due to the limited availability and greater competition for synchrotron time compared to the wider availability of lab-based instruments.

Improvements in image quality not only provide better statistics when analyzing electrode level degradation but also enable higher quality segmentation with the possibility of identifying individual particles. Image analysis techniques that were once largely within the domain of ex-situ electrode scans can now be conducted on the same sample volume as a function of variables including SoC and cycle number. Thus, parameters that can be quantified in-situ include active material loading, active material distribution within the electrode, contact area between the solid phases, and pore and particle size distributions. Furthermore, degradation phenomena occurring at the electrodes can be investigated at various length scales, which improves the understanding of their influence on electrochemical performance and lifetime.

**Figure 9:**
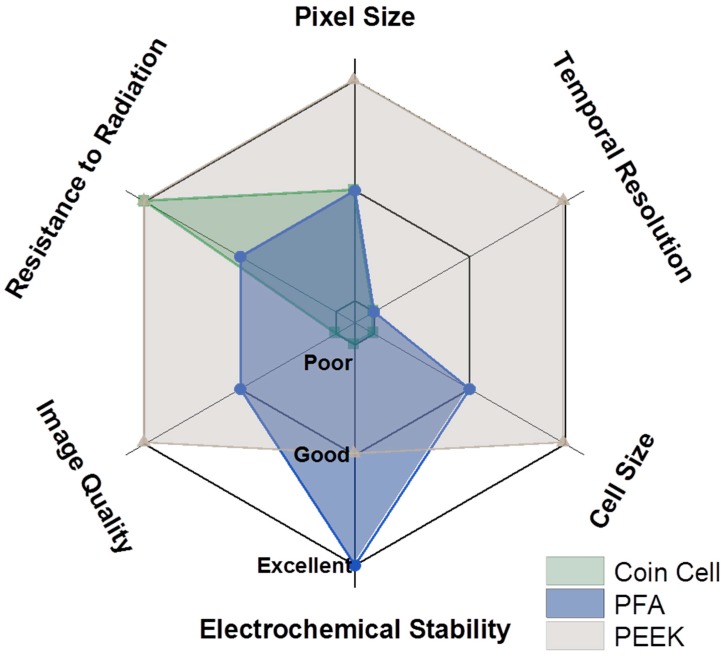
Comparison of the various iterations of cell designs.

The considerations presented throughout and summarized in Figure 9 indicate that reducing the size of the cell is paramount to decreasing the total scan time while considerably improving both spatial and temporal resolutions and image quality.

### 2.8. Other Avenues for Improvement and Future Work

In addition to the parameters considered here, other avenues for improvement include maintaining constant compression within the cell and controlling the electrolyte volume during initial cell filling. It has been reported extensively that cell compression [30] and insufficient electrolyte can influence the electrochemical performance of a cell [31]. It is, therefore, desirable to establish a method that allows constant contact pressure between the electrodes and the current collectors and sufficient electrolyte volume. Future designs may look to incorporate spring loading into the cell assembly and vacuum filling of electrolyte to further optimize the in-situ cell. 

Another avenue for improvement may come during tomographic reconstruction since the widely used filtered back-projection algorithm requires a high angular resolution for successful reconstruction. Iterative reconstruction methods that employ intelligent algorithms to reduce the number of projections required for an equivalent reconstruction may be beneficial in the study of electrochemical devices. Many iterative reconstruction algorithms exist but they are typically based around the same method. A forward projection of an estimate creates artificial data which is compared to the real raw data that is collected from the sample. From this comparison, a correction term is computed that is back-projected onto the estimate. These iterative reconstruction computations conclude after a certain number of iterations or once the difference between the estimate and the raw data (known as the ‘update’) converges to a sufficiently small value.

Iterative reconstruction methods based upon the improvement of either individual pixels, the entire projection, or a subset are known as the Algebraic Reconstruction Technique (ART), the Simultaneous Algebraic Reconstruction Technique (SART), or the Ordered Subset (OS) method, respectively. All methods are additive in nature, based on the addition of the update onto the current solution, and OS-based methods are typically the fastest even though they may be susceptible to artefacts. Multiplicative methods also exist such as the Multiplicative Algebraic Reconstruction Technique (MART).

The use of iterative reconstruction techniques may permit higher temporal resolution studies, which are particularly of interest for studies involving highly dynamic mechanisms such as thermal runaway [32] and rapid charging of batteries [33]. For more information on iterative reconstruction techniques, the reader is directed to work by Beister et al. [34].

## 3. Conclusions

As research into novel cell chemistries has expanded over the past few decades, it is clearly of great scientific interest to visualize the electrodes of electrochemical cells at all stages in their developmental lifecycle to extract performance gains and maximize their capacity, power, and lifetime. Through in-situ and operando X-ray tomography, electrode microstructures can be visualized in 3D as a function of variables such as state of charge and cell age and conditions such as thermal abuse or overcharging. We believe that in-situ and operando tomography will ultimately achieve a similar impact to X-ray diffraction and spectroscopy in the design and engineering of new battery materials.

In addition to qualitative observations on electrode degradation, techniques such as digital volume correlation can be used to track and quantify microstructure evolution chronologically within the electrodes. With a better understanding of the fundamental processes occurring at the electrodes and how cell conditions and configurations affect these processes, electrode materials can be optimized to improve their lifetime and performance. The improvements in spatial and temporal resolutions gained from the use of optimized cell designs for micro-CT far outweigh the impact on electrochemical performance these designs may have. 

## 4. Methodology

### 4.1. Electrode Preparation

For the Li-ion cell electrodes, LiNi_0.33_Mn_0.33_Co_0.33_O_2_ (NCM111, MTI Corp., St. Louis, MO, USA) or LiMn_2_O_4_ (LMO, MTI Corp.), conductive carbon black (Super C65, Imerys, Paris, France), and polyvinylidenefluoride (PVDF) (Solef 5130, Solvay, Brussels, Belgium) in a 90:5:5 mass ratio were homogenized with a planetary centrifugal mixer (ARE-250, Thinky Corporation, Tokyo, Japan) using anyhdrous *N*-methyl-2pyrrolidinone (NMP, Sigma Aldrich, Saint Louis, MO, USA) as solvent. The slurry was cast on a 15 μm thick aluminium foil (MTI Corp.) using a micrometre adjustable film applicator set to a blade gap of 150 μm.

For the Li-S cell, elemental sulfur (325 mesh, Alfa Aesar, Haverhill, MA, USA), conductive carbon black (Super C65, Timcal, Bodio, Switzerland), Ketjenblack (EC600-JD, Akzo Nobel, Amsterdam, The Netherlands), and polyvinylidene fluoride binder (Solef 5130, Solvay) in a 75:12:3:10 mass ratio were homogenized with a high shear laboratory mixer (L5M, Silverson, Buckinghamshire, UK) to form an ink with 20% total solids content with anhydrous NMP as solvent. The ink was cast onto 15 μm thick aluminium foil using a micrometre adjustable film applicator set to a blade gap of 400 μm.

The electrode sheets were initially dried on a hot plate at 80 °C and subsequently dried overnight under vacuum at 120 °C (for Li-ion electrodes) or 60 °C (for S electrode). Additionally, 3.15 mm and 0.77 mm disks were cut from the sheets by using a laser micro-machining instrument (A Series Compact Micromachining System, Oxford Lasers Ltd., Didcot, UK).

### 4.2. Coin Cell with Kapton Window 

CR2032-type coin cell (CR2032, MTI) were cleaned in isopropanol (≥99.5% purity) and dried overnight under vacuum at 60 °C prior to use. A 16 mm wide × 3 mm high letterbox-shaped aperture with a 6 mm diameter circular hole in the centre was drilled into the can, cap, and spacer components of the coin cells. Rectangular strips (ca. 19 mm × 5.5 mm) of 50 μm thick adhesive Kapton tape were applied on both the internal and external surfaces of the coin cell cap and can components to create an X-ray transparent window. Epoxy adhesive (Araldite) was then applied over the edges of the external Kapton strips to create a hermetic seal around both windows. The circular hole in the centre of the coin cell was designed to aid in sample alignment during tomography scans and the letterbox shaped portion of the X-ray window was designed to provide a sufficiently large angular range for tomographic acquisition while maintaining the mechanical stability of the coin cell.

This window design meant that tomographic acquisition performed with the in-situ coin cells were limited angle scans with the angular range dependent on the field of view. During coin cell assembly, the X-ray windows on the cell casings and spacer components were carefully aligned in order to avoid beam attenuation by the dense metal components, which ensures a clear ‘line-of-sight’ for the X-ray beam being transmitted through the electrode material. All coin cells were assembled with the LMO electrode as a working electrode, glass fiber separator soaked in LiPF_6_-based electrolyte and a metallic lithium counter electrode.

### 4.3. 1/8” PFA Swagelok

The 3.15 mm electrode disks were dried in a transferrable vacuum oven (Glass Oven B-585 Drying, Buchi, Flawil, Switzerland) at 120 °C (NMC electrode) or 60 °C (S electrode) overnight and transferred to an argon filled glovebox (MBraun, LABstar, Garching, Germany) where both O_2_ and H_2_O levels were maintained below 0.5 ppm. Customized 1/8” PFA Swagelok unions (PFA-220-6, Swagelok, Soren, OH, USA) were used as cell bodies and these were assembled using 1/8” 316L stainless steel plungers as the current collector. Excess material was removed from the centre of the PFA union to reduce the X-ray attenuation. Lithium metal punched to 1/8” was used as the counter electrode with glass fiber punched to 4 mm (GF/D, Whatman, Maidstone District, UK) as a separator. For Li-ion cells, 1.2 M lithium hexafluorophosphase (LiPF_6_) in ethylene carbonate and ethyl methyl carbonate (EC:EMC, 1:2 *v*/*v*, Soulbrain, Northville Township, MI, USA) was used as an electrolyte. For Li-S cells, 1 M lithium bis(trifluoromethane) sulfonimide (LiTFSI) in 1,3-dioxolane and 1,2-dimethoxyethane (DOL:DME, 1:1 *v*/*v*) with 0.3 M lithium nitrate as an additive (Soulbrain, Northville Township, MI, USA) was used as an electrolyte.

### 4.4. 1/32” PEEK Union

The 0.77 mm electrode disks were dried in a transferrable vacuum oven (Glass Oven B-585 Drying, Buchi) at 120 °C (NMC electrode) or 60 °C (S electrode) overnight and transferred to an argon filled glovebox (MBraun, LABstar, Garching, Germany) where both O_2_ and H_2_O levels were maintained below 0.5 ppm. Bespoke 1/32” polyether ether ketone (PEEK) unions were used as cell bodies for the miniature tomography cells with 316L stainless steel plungers as the current collector.

Lithium metal punched to 0.8 mm was used as the counter electrode with glass fiber punched to 1 mm (GF/D, Whatman) as a separator. For Li-ion cells, 1.2 M lithium hexafluorophosphate (LiPF_6_) in ethylene carbonate and ethyl methyl carbonate (EC:EMC, 1:2 *v*/*v*, Soulbrain, Northville Township, MI, USA) was used as an electrolyte. For Li-S cells, 1 M lithium bis(trifluoromethane) sulfonimide (LiTFSI) in 1,3-dioxolane and 1,2-dimethoxyethane (DOL:DME, 1:1 *v*/*v*) with 0.3 M lithium nitrate as an additive (Soulbrain, Northville Township, MI, USA) was used as an electrolyte.

### 4.5. Synchrotron Micro-CT Acquisition and Reconstruction

Synchrotron micro-CT was performed at the i13-2 beamline at Diamond Light Source (Harwell, UK) in the absorption contrast imaging mode. A parallel beam was used for the interior tomography of an LMO electrode sample assembled within the in-situ coin cell. The incident X-ray beam was monochromatized to 16 keV by a water-cooled double crystal Si <111> monochromator. The sample to detector distance was set to 25 mm and an average useful rotation range of 147° was achieved through the Kapton window. Projection images were acquired when the sample was rotated through angular steps of 0.1° about its long axis with a 6 s exposure time per projection. A 9.6 μm thick GGG:Eu scintillator was coupled to a 10× objective lens and projections were captured with a 2000 × 2000 pixel pco4000 CCD detector, which resulted in an effective pixel size of 0.365 μm.

### 4.6. Laboratory Micro-CT Acquisition and Reconstruction

X-ray micro-CT was performed on the PFA and PEEK in-situ cells with a lab-based micro-CT instrument (Zeiss Xradia Versa 520, Carl Zeiss Inc., Oberkochen, Germany). The instrument consisted of a polychromatic micro-focus sealed source set to an accelerating voltage of 80 kV on a tungsten target at a maximum power of 7 W. The scintillator was coupled to either a 20× or 40× objective lens and 2048 × 2048 pixel CCD detector with a pixel binning of 1, which results in a pixel size of ca. 460 nm and a field of view of ca. 940 μm for the 20× objective and ca. 230 nm and a field of view of ca. 470 μm for the 40× objective. There was no significant geometric magnification since the sample was set close to the detector to reduce the effects of penumbral blurring arising from the cone beam nature of the source. The sample was rotated through 360° with radiographs collected at discrete angular intervals amounting to a total of 1601 projections. The radiographic projections were then reconstructed with proprietary reconstruction software (Version 11.1.8043, XMReconstructor, Carl Zeiss Inc.) by using a modified Feldkamp-David-Kress (FDK) algorithm for cone beam geometry.

## Figures and Tables

**Table 1:** Comparison of common materials used in construction of Swagelok-type cells.

Material	Moisture Impermeability	Li-ion Electrolyte Compatibility	Reactivity with Li	X-ray Transparency and Compatibility
Stainless Steel	Excellent	Some grades are compatible	Non-reactive	Poor
Aluminium	Excellent	May be corroded by electrolyte	Alloys with Li	Good
Beryllium	Excellent	May be corroded by electrolyte	Non-reactive	Excellent
Polyimide (Uncoated)	Poor	Compatible	Non-reactive	Excellent
PFA	Good	Compatible	Reacts with metallic Li to form elemental carbon	Transparent but susceptible to radiation damage
PTFE	Good	Compatible	Reacts with metallic Li to form elemental carbon	Transparent but susceptible to radiation damage
PEEK	Good	Compatible	Non-reactive	Good
